# Spatially Resolved Distribution, Sources, Exposure Levels, and Health Risks of Heavy Metals in <63 μm Size-Fractionated Road Dust from Lucknow City, North India

**DOI:** 10.3390/ijerph191912898

**Published:** 2022-10-08

**Authors:** Vidhu Gupta, Lalita Bisht, Ajay Kumar Arya, Ajay Pratap Singh, Sneha Gautam

**Affiliations:** 1 Department of Environmental Sciences, Hemvati Nandan Bahuguna Garhwal University, Srinagar 246174, Uttarakhand, India; 2 Department of Geology, University of Lucknow, Lucknow 226007, Uttar Pradesh, India; 3 Department of Civil Engineering, Karunya Institute of Technology and Sciences Coimbatore, Coimbatore 641114, Tamil Nadu, India

**Keywords:** road dust, heavy metals, pollution assessment, spatial distribution, potential ecological risk, health risk assessment

## Abstract

In the present study, a total of 64 road dust samples were collected from five different functional areas (residential, commercial, parks, high-traffic, and industrial) in urban Lucknow to assess the accumulation, distribution, and health risk of heavy metals (HMs) (i.e., Fe, Mn, Zn, Cu, Pb, Cd, As, Cr and Ni). Acid digestion methods were used to analyze HMs, followed by inductively coupled plasma-mass spectrometry (ICPMS). The ascending frequency of HMs was Cd < As < Ni < Cr < Pb < Cu < Zn < Mn < Fe for all different functional areas. Almost all HMs exceed the limits of Indian natural soil background values (INSB) across all functional areas. The pollution assessment results reveal that the urban road dust of Lucknow is highly enriched with Zn and Pb, causing deterioration of dust quality. The spatial distribution of HMs shows that road dust found in the central and southwestern zones of the Lucknow urban area are more contaminated than in other areas. The ecological risk assessment demonstrates that Cd was the highest risk contributor, followed by Pb, Zn and Cu. The result of the health risk assessment i.e., the cumulative hazard index (HI) and the cumulative lifetime cancer risk (LCR), reveal that children (mean HI_children_ = 1.26, LCR_children_ = 0.000187) are more vulnerable to HM exposure than adults (HI_adults_ = 0.14, LCR_adults_ = 0.0000804). For carcinogenic and non-carcinogenic risk, ingestion appears to be the major pathway of HM exposure in both age groups. It is alarming that all studied four carcinogenic HMs were found in concentrations higher than 1 × 10^−6^ (the permissible limit for humans). This indicates slight chances of developing cancer for both age groups in all functional areas.

## 1. Introduction

Dust particles are ubiquitous. No one is untouched from dust exposure, indoors or outdoors. Apart from natural sources, dust particles are produced from various anthropogenic sources in urban areas, such as fossil fuel burning, vehicular emissions, industrial operations, waste incineration, construction, demolition, and weathering of road surface material [1,2,3,4]. Road dust particles are mainly generated via road-related activities and are loaded with various inorganic and organic compounds [5]. HMs are one of the most dangerous substances due to their non-biodegradability, accumulation in other environmental components, and ability to create toxicity in humans [6]. Vehicular-fuel-burning and non-fuel-burning activities are primarily responsible for HMs in road dust. HMs in road dust are primarily linked with vehicle exhaust and non-exhaust emissions [7,8]. Vehicular exhaust emissions include petrol and diesel burning and the non-exhaust category includes wear and tear of tires and brake pads, corrosion of batteries and metallic parts, and road surface degradation. Emissions from nearby industries, incineration activities, solid waste disposal, and construction and demolition activities also play an important role in increasing HMs in an urban environment [5,9,10]. Various meteorological factors such as temperature, wind direction and velocity, frequency and intensity of rain, and preceding dry periods influence the deposition, distribution, and concentration of HMs in road-deposited dust. Other factors such as the particle size of the pollutant, vegetation cover, population density, traffic volume, road surface conditions, highway or road design, and the material of road surfaces also affect the concentration and distribution of HMs in the dust deposited on road surfaces [4,11,12].

Road dust particles contain several chemical components including HMs. Human beings are exposed to road dust via ingestion through the mouth, inhalation via the nose, and dermal contact or absorption of dust particles with skin [3,13]. Dust contaminated with HMs can pose serious threats to human health and the environment. Excessive intake of HMs can lead to neurological and reproductive system dysfunction, gastrointestinal issues, kidney failure, and cardiovascular as well as bone diseases [13,14,15]. Even at very low concentrations, HMs can accumulate in human internal organs and can cause health hazards. Pb and Zn are toxic to the central nervous system. Overexposure to Fe and Mn can cause neurological disorders, cardiovascular disease, Parkinson’s disease, and Huntington’s disease [16,17]. Pb is often associated with low IQ and can affect children’s behaviors and create learning issues in children [18,19,20,21]. Pb and As can cause damage to the central nervous system and the immunological system, and can cause renal dysfunction, dermal lesions, skin cancer, and hypertension [14,22,23,24]. Long-term exposure to HM-enriched dust can also cause respiratory problems, especially in asthmatic people [25,26]. Continuous exposure to HMs such as Cd, As, Ni, and Cr can be carcinogenic to respiratory organs and cause human lung cancer [27].

Although numerous studies [2,13,22,28,29,30,31,32,33] on HM contamination of road dust have been carried out around the world, very little information is available for countries such as India, where a huge percentage (17.7%) of the total world population lives (Census of India, 2011). However, most studies [5,6,8,23,34] have focused only on concentration levels and pollution status, and information regarding spatial distribution and health in different functional areas is very limited [3,35,36]. Rapid urbanization has transpired in the Lucknow district over the previous decade, particularly in the Lucknow urban area, resulting in high population and vehicular density in the city, which has also increased pollution levels in the city (Ministry of Road Transport & Highways, Government of India). Therefore, the present study primarily focuses on the following objectives: (1) Determining the concentration and spatial distribution of HMs in road dust collected from different functional areas in the Lucknow urban area; (2) Presenting the source appointment for different HMs in road dust using principal component analysis; (3) Evaluating and assessing HM pollution in different functional areas in road dust over the Lucknow urban areas; (4) Quantifying ecological and human health risk assessment. The present study aims to establish baseline data on HM concentration associated with road dust, ultimately influencing air quality and human health quality in urban areas. Moreover, the main focus of the study is allowing government officials and the general public to gain insight into the toxicity and health risks of metal-bound dust, leading to better understanding of how to control them.

## 2. Materials and Methods

### 2.1. Study Area

**Figure 1:**
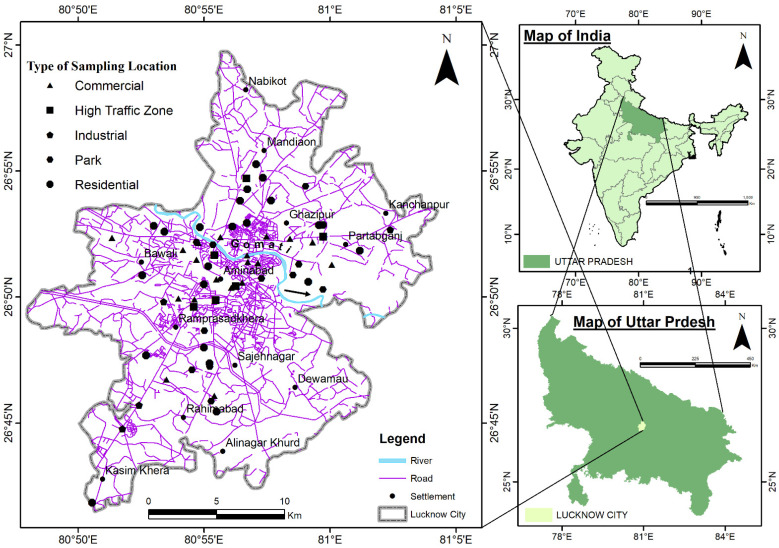
Map of the study area and sampling locations.

The present study was carried out in Lucknow, an urban area, which is the capital city of Uttar Pradesh (Figure 1). Figure 1 presents a map of Lucknow indicating 64 sampling locations representing five different functional areas i.e., residential (N = 23), commercial (N = 19), high-traffic areas (N = 6), parks (N = 12), and industrial areas (N = 4) throughout the study area. The city lies at 80°56’ E longitude and 26°30’ N latitude and is 128 m above sea level. It has a total area of approximately 310 km^2^. Good infrastructure, the presence of various other amenities, and well-connected routes of transportation make Lucknow the second-largest urban/metropolitan city of India with a population of 2,902,290, of which 1,509,451 are males and 1,393,469 are females (Census, India 2011). Due to the large population and well-connected road system, Lucknow experiences high vehicular flux throughout the year. The total registered vehicular population in Lucknow was 2,194,261 in 2018–2019 and 2,407,190 in 2019–2020, which is a 9.70% growth over the previous year (Source: Regional Transport Office, Lucknow).

### 2.2. Sample Collection and Chemical Analysis

A total of 64 road dust samples were collected under stable weather conditions in the cold and dry seasons during the month of January 2020. All sampling locations are mentioned in Table 1. From each sampling location, 2–3 sub-sites were chosen for road dust collection. Approximately 400–500 g of dust was collected from each sampling location with a small clean plastic brush and a dustpan. Dust was collected from the middle and sides of the road in each sampling site. After that, the sample was transferred to the laboratory in labeled self-sealing polythene bags. In the laboratory, road dust samples were air-dried and sieved with a 2 mm mesh to remove large particles. The remaining dust was again sieved using a stainless-steel sieve with a 63 μm mesh size. A smaller size fraction (<63 μm) of road dust was used for HMs analysis because of their affinity to absorb a higher amount of HMs which can cause more health hazards and remain suspended in the air for a prolonged time [3,8].

The HMs concentration in the road dust was determined by the acid digestion method as prescribed by USEPA 3050B [37]. A total of 1 g of dried homogenized road dust was digested with a 3:1 mixture of 9 mL nitric acid (HNO_3_) and 3 mL hydrochloric acid (HCl) on a hot plate. The solution was heated on the hotplate at 95 °C ± 5 and allowed to evaporate with boiling until 1–2 mL of solution remained. To minimize the effervescence, 2 mL of hydrogen peroxide (H_2_O_2_) was added. Following digestion, the sample was cooled down and filtered through a Whatman Filter (No.42) in a pre-marked test tube and the resulting solution was then diluted up to 15 mL using deionized water. The HMs were analyzed in the samples using inductively coupled plasma-mass spectrometry (ICP-MS, Perkin Elmer, Make: Agilent, Model no. NexION 300X) [3,4,37].Prior to analysis, the instrument was calibrated for each metal using a series of known standards. Different series of working standards were prepared for different HMs through serial dilution using a multi-element stock solution of 1000 ppm (Inorganic Venture IV-ICPMS-71A). For each HM, a different series of working standards were prepared. Reagent blanks and duplicate samples were used as quality control for the strong acid digestion procedure. Type-I Milli-Q ultra-pure water was used to prepare solutions, dilutions, and blanks throughout the procedures. All utensils used in the analysis were cleaned with 10% v/v HNO_3_ followed by ultra-pure deionized water before usage [3].

### 2.3. Pollution Assessment

Two different methods were used to evaluate the pollution levels for HMs in the dust: (i) pollution index (PI) and integrated pollution index (IPI) and (ii) geo-accumulation index (I_geo_).

PI and IPI values indicate the function of HMs and are used to assess HM contamination in road dust [38]. The PI for each HM was determined by dividing each metal’s HM concentration by the respective HM’s background value [28].

(1) $$\mathrm{PI}=\frac{C}{B}$$

where C represents the HM concentration and B represents the HM background values. 

The IPI is the mean value of the PIs of HM studied in the road dust.

(2) IPI=PI1+PI2+. . . . . . . . . . . . . . . . PInn

where PI indicates the HM’s pollution index and n indicates the total number of studied HMs.

The PI and IPI of all HMs were classified into three and four categories, respectively, which are shown in Table 2.

The geo-accumulation index (I_geo_) index wasp roposed by Muller [39] and has been widely used to assess the pollution level of heavy metals in road dust [33,40,41]:

(3) Igeo=Log2Ci1.5×Bi

where C_i_ is the concentration of i HM and B_i_ is the background value of i HM. The textural matrix modification factor due to lithosphere impacts is constant i.e., 1.5 [28,41]. The I_geo_ pollution level is categorized into seven categories as follows: I_geo_ < 0 (unpolluted); 0 ≤ I_geo_ ≤ 1 (unpolluted to moderately polluted); 1 ≤ I_geo_ ≤ 2 (moderately polluted); 2 ≤ I_geo_ ≤ 3 (moderately to strongly polluted); 3 ≤ I_geo_ ≤ 4 (strongly polluted); 4 ≤ I_geo_ ≤ 5 (strongly to extremely polluted); and I_geo_ ≥ 5 (extremely polluted).

The Indian natural soil background values (INSB) [42,43] were adopted as background values to calculate the pollution indices (PI and I_geo_),because the background values of HMs in road dust were unavailable.

### 2.4. Statistical Analysis

The statistical analysis was carried out using Microsoft Excel (Version 7, Microsoft Corporation, Washington, DC, USA). Statistical Package for the Social Sciences (IBM SPSS Inc. Version 22.0, New York, NY, USA) was used to conduct principal component analysis (PCA) to determine the possible sources of HMs in road dust.

### 2.5. Spatial Distribution

The concentrations of nine HMs (Fe, Mn, Zn, Ni, Cu, Pb, As, Cr, and Cd) were mapped to analyze the distribution of HMs across Lucknow city and to identify the pollution-sensitive areas by an inverse distance weighting (IDW) interpolation method through Arc GIS software (Version 10.4, ESRI, Readlands, CA, USA).

### 2.6. Risk Assessment

#### 2.6.1. Potential Ecological Risk (PER)

The potential ecological risk index (PER) was developed by Hakanson [44] to assess the risk levels of HMs in road dust particles [5,33,44,45]. PER was calculated using the following formula:

PER = ∑E_r_ = ∑T_r_ × PI (4)

where E_r_ is the ecological risk factor of HM and T_r_ is the toxic-response factor of HM (Table 3) As the toxic response factor of Fe is not available, only eight metals are included in the PER study. PI is the pollution index of the HM. Hakanson [44] classified E_r_ and PER into five and four potential risk categories, respectively, which are given in Table 2.

#### 2.6.2. Health Risk Assessment

Health risk assessment (HRA) was performed using the health risk framework model developed by the United States Environmental Protection Agency [46] for both children and adults for HMs in road dust samples collected from different functional areas. The non-carcinogenic risk was calculated for all studied HMs, but the carcinogenic risk was calculated only for class 1 carcinogenic heavy metals i.e., Cd, As, Cr, and Ni, [47]. Human exposure to HMs mainly happens through three exposure routes i.e., ingestion, inhalation, and dermal contact, which were calculated in the form of the average daily intake (ADI) for both children and adults using the following formulas:

(5) $$\mathrm{ADIingestion}\;=\;C\;\times \frac{\mathrm{IngR}\times \mathrm{ED}\times \mathrm{EF}}{\mathrm{BW}\times \mathrm{AT}}\times 10^{-6}$$

(6) $$\mathrm{ADIinhalation}=\;C\;\times \frac{\mathrm{InhR}\times \mathrm{ED}\times \mathrm{EF}}{\mathrm{PEF}\times \mathrm{BW}\times \mathrm{AT}}$$

(7) $$\mathrm{ADIdermermal}=\;C\;\times \frac{\mathrm{SL}\times \mathrm{ABS}\times \mathrm{SA}\times \mathrm{ED}\times \mathrm{EF}\;}{\mathrm{BW}\times \mathrm{AT}}\times 10^{-6}$$

Table 3 lists the exposure factors used in ADI evaluation to assess the non-carcinogenic risk for children and adults based on USEPA [46].

The non-carcinogenic risk for both individuals via three different exposure pathways for each studied HM was assessed by calculating their respective HQ values, which are equal to the average daily dose divided by the reference dose of the corresponding HM [5,48]. Table 4 shows the values of the reference doses for studied HMs based on the US Environmental Protection Agency (USEPA) [49]. The cumulative non-carcinogenic risk, also known as hazard index (HI), via three exposure routes was calculated by adding their respective HQs (HI = HQ_ing_ + HQ_inh_ + HQ_derm_). According to USEPA [46], a HI value less than 1 (HI > 1) indicates that there is no significant adverse non-carcinogenic risk, whereas a HI value of more than 1 (HI < 1) indicates the possibility of non-carcinogenic risk in both children and adults [5,50].

The carcinogenic risk assessment for Cd, As, Cr and Ni in the dust samples was carried out by calculating the cancer risk (CR) and the lifetime cancer risk (LCR). CR is the ratio of the HMs average daily dose and cancer slope factor (CSF). The summation of the total cancer risk of a HM via three exposure routes (LCR = Cancer risk_ing_ + Cancer risk_inh_ + Cancer risk_derm_) is termed the lifetime cancer risk (LCR). CSF values for Cd, As Cr, and Ni are given in Table 4. For regulatory purposes, the tolerable range of cancer is 10^−4^ to 10^−6^ [49,50,51].

## 3. Results

### 3.1. Heavy Metal Concentration in Road Dust

Table 5 summarizes the descriptive analysis of HM concentrations in road dust measured in various functional areas in Lucknow city, India. The overall average HM concentrations in the road dust samples were 16,368.15 μg·g^−1^ for Fe, 374.15 μg g^−1^ for Mn, 289.85 μg g^−1^ for Zn, 139.66 μg g^−1^ for Cu, 97.34 μg g^−1^ for Pb, 1.29 μg g^−1^ for Cd, 8.82 μg g^−1^ for As, 87.29 μg g^−1^ for Cr, and 54.23 μg g^−1^ for Ni. Concerning mean concentration, the ascending order of HMs was Cd < As < Ni < Cr < Pb < Cu < Zn < Mn < Fe for all different functional areas. As there are no regulatory standards for HMs in road dust in India and other countries, the results were compared with the Indian natural soil background (INSB) values [42,44] and earth’s upper continental crust (UCC) [52] values (Table 6). The mean concentration of Zn, Pb, Cu, Mn, Ni, and Cd were 13.1-, 7.4-, 2.4-, 1.7-, 1.09-, and 1.40-times higher than the INSB values, respectively, whereas Fe and Cr were lower than the INSB values. The value of As concentration is not given in the INSB. The mean concentration of all studied HMs was greater than the UCC background values, except for Fe. The average concentration of Zn, Pb, Cu, Cr, Mn, Ni, and Cd were 5.5-, 5.7-, 9.7-, 2.4-, 1.7-, 2.9-, and 12.6-times higher than the UCC values, respectively. This shows that anthropogenic sources contribute a significant amount of HMs to the road dust in the urban environment of Lucknow.

The results of the present study are comparable to the observations of various cities in India and other countries (Table 6). It is seen that the levels of HMs (Fe, Mn, Zn, Cu, As, and Ni) in Lucknow are higher than in Dehradun (India), Xi’an (China), and Dezful (Iran). The level of Cr is higher than Dhanbad and Kolkata (India), Dhaka (Bangladesh), and Lahore (Pakistan), and lower than Delhi (India) and Kabul (Afghanistan). The level of Pb is comparable to Xi’an (China) and higher than Dhaka (Bangladesh) and Dezful (Afghanistan), but lower than Delhi, Dhanbad, Kolkata, and Dehradun (India), Lahore (Pakistan), and Kabul (Afghanistan). With respect to the functional areas, all studied nine HMs were found to be highest in the industrial areas, followed by high-traffic areas and commercial areas, and were lowest in residential and park zones. These results show similar trends to other functional area studies e.g.,Suryawanshi et al. [8] in Delhi (India), Kolakkandi et al. [36] in Kolkata (India), Wei et al. [53] in Beijing (China), and Rehman et al. [18] in Dhaka (Bangladesh), where industrial and high-traffic zones show the higher concentration of metals than other functional are as such as residential areas and parks. This is mainly due to high traffic densities and different industrial activities.

### 3.2. Pollution Assessment of Heavy Metals in Road Dust

**Figure 2:**
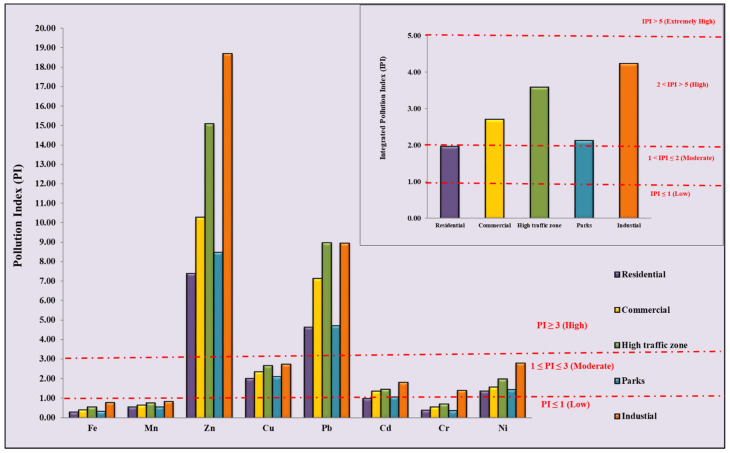
Pollution indexes (PIs) and integrated pollution indexes (IPI) of HMs in the dust of Lucknow over different functional areas.

The pollution assessment in different functional areas for the eight studied HMs is presented in Figure 2. Due to the unavailability of a background value of As in the INSB, the pollution assessment of As is not calculated. The PI values show that Zn and Pb fall in the high pollution level category in all functional areas. The average PI values show that Cu, Cd, and Ni come under the moderate pollution level category, while Cr, Fe, and Mn come under the low pollution level category. The descending order of PIs of studied HMs is Zn > Pb > Cu > Ni > Cd > Cr > Mn > Fe. The descending order of HM accumulation in different functional areas isas follows: industrial > high-traffic zone > commercial > parks > residential areas. Average IPI values show a high level of pollution (2 ≤ IPI ≤ 5) in all functional areas. This confirms that the sources of Zn, Pb, Cu, Cd, and Ni were ubiquitous over urban Lucknow and mainly come from traffic emissions and industrial activity. Cr, Fe, and Mn come from mixed sources and their geogenic contribution is significant for these three HMs. The results of the present study show that out of a total of 64 sampling sites, 71.88% of sites fall into the high pollution category (2 ≤ IPI ≤ 5), 26.56% of sites fall into the moderate pollution category (1 ≤ IPI ≤ 2) and 1.56% of locations fall into the extremely high pollution category (IPI > 5), indicating the high pollution level in the Lucknow urban area.

**Figure 3:**
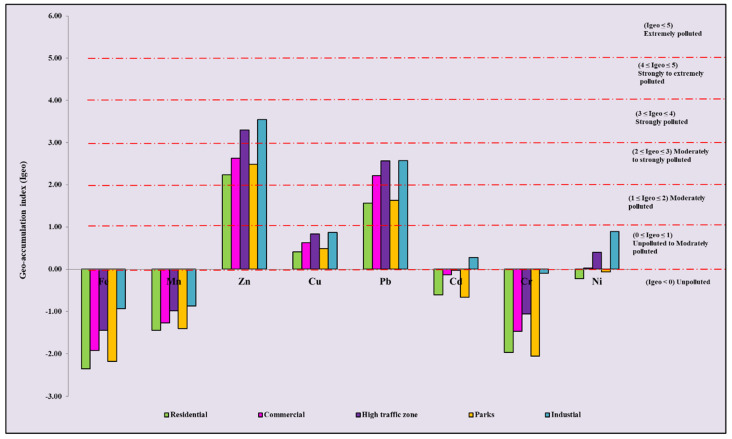
Geo-accumulation index (I_geo_) of HMs in the road dust of Lucknow over different functional areas.

Figure 3 shows the I_geo_ values of HMs in all five studied functional areas. The descending order for the average I_geo_ values is as follows: Zn > Pb > Cu > Ni > Cd > Mn > Cr > Fe. The value of Zn shows that the road dust of industrial and high-traffic areas is heavily polluted, whereas commercial, parks, and residential areas are moderately to heavily polluted with Zn. Pb values indicate moderate pollution levels in residential and parks areas, and moderate to heavy pollution levels in commercial, high traffic, and industrial areas. In all different functional areas, Cu showed an unpolluted to moderate pollution level. In industrial, commercial, and high-traffic areas, Ni values indicate unpolluted to moderate pollution levels, but in residential and park areas, Ni values indicate low pollution. Cd only shows unpolluted to moderate pollution in industrial areas and low pollution in other functional areas. Cr, Fe, and Mn are in the unpolluted category in all different functional areas. It is seen that the industrial areas were the most polluted among the five functional areas, followed by high-traffic areas; Zn and Pb are the most common HMs among the studied HMs.

### 3.3. Source Appointment of Heavy Metals in Road Dust

PCA was used to appoint HM sources in the road dust across the study areas. Table 7 summarizes the PCA results, including PCA component factors, eigenvalues, percentage variance, and the cumulative percentage variance. The PCA data showed three main components with an eigenvalue greater than 1, accounting for 84.41% of the total variance. The first component was dominated by Mn, Zn, Cu, and Pb, which account for 33.66% of the total variance and represent the anthropogenic source of origin, which could be vehicular emissions (exhaust and non-exhaust sources). The second component was dominated by Fe, Cr, and Ni, accounting for 27.55% of the total variance. These metals come into road dust via both sources i.e., anthropogenic and natural. Component 3, which accounts for 23.30% of the total variance, was dominated by Cd and As and represents the industrial source.

### 3.4. Spatial Distribution of Heavy Metals (Concentration) over the Lucknow Urban Area

**Figure 4:**
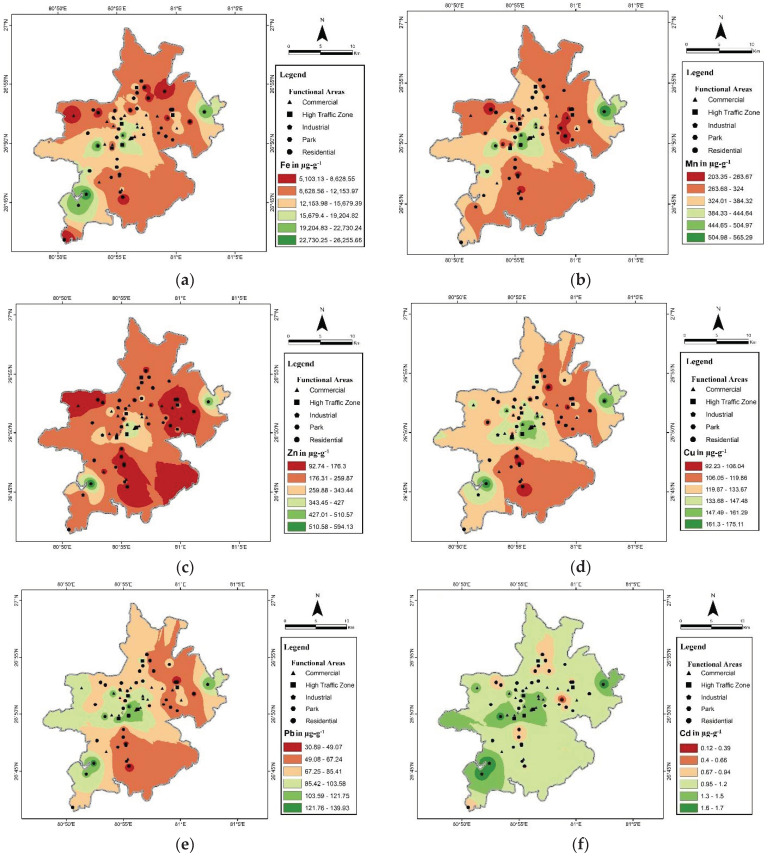

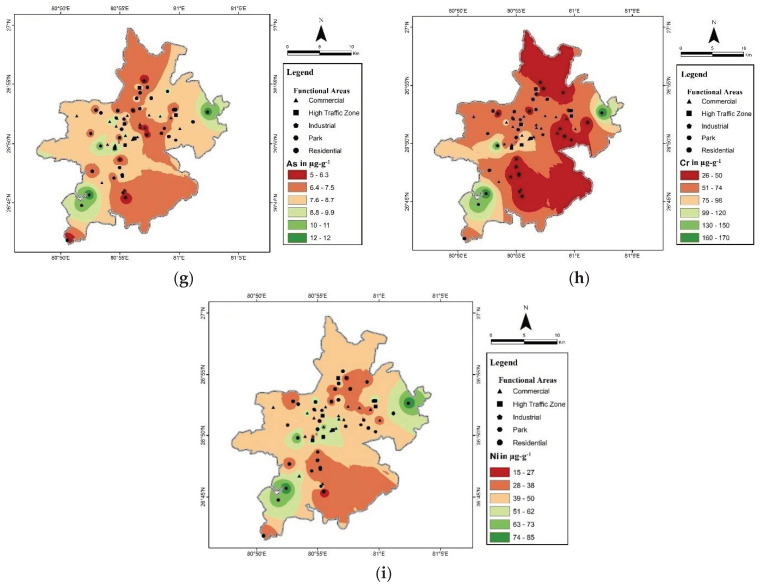
(**a**–**i**) Spatial distribution of HMs in the road dust of Lucknow over different functional areas.

The spatial distribution maps of HM concentration are displayed in Figure 4a–i. The center of the city and the southwestern zone showed a high concentration range for almost all the studied HMs, because major commercial centers, high-traffic zones and industrial locations i.e., Talkatora are, are located in these regions. The far northeastern region showed a high concentration of HMs because of the densely populated Chinhat industrial area. Southeastern and north locations were in the lower concentration range of the metals as compared to other locations. These zones consist of residential areas and parks, and have lower traffic density.

Vehicular-generated HMs such as Zn and Cu show higher range over a large area because traffic density is higher in all the zones except the outer peripheral zone and the southeastern zone, which are dominated by residential areas and the cantonment zone. The industrial zone (found in the southwestern and far eastern zone) also shows the highest concentration range compared to other zones for HMs such as Cr, Ni, Pb, and Cd. Some metals specifically emitted from industrial activities, such as Cd, As, Ni and Cr, were found highest around the center of the city as shown on the map; however, concentration levels appear to be similar in most of the areas, implying that natural processes also influence the concentration of these metals in study areas.

### 3.5. Risk Assessment

#### 3.5.1. Potential Ecological Risk (PER)

**Figure 5:**
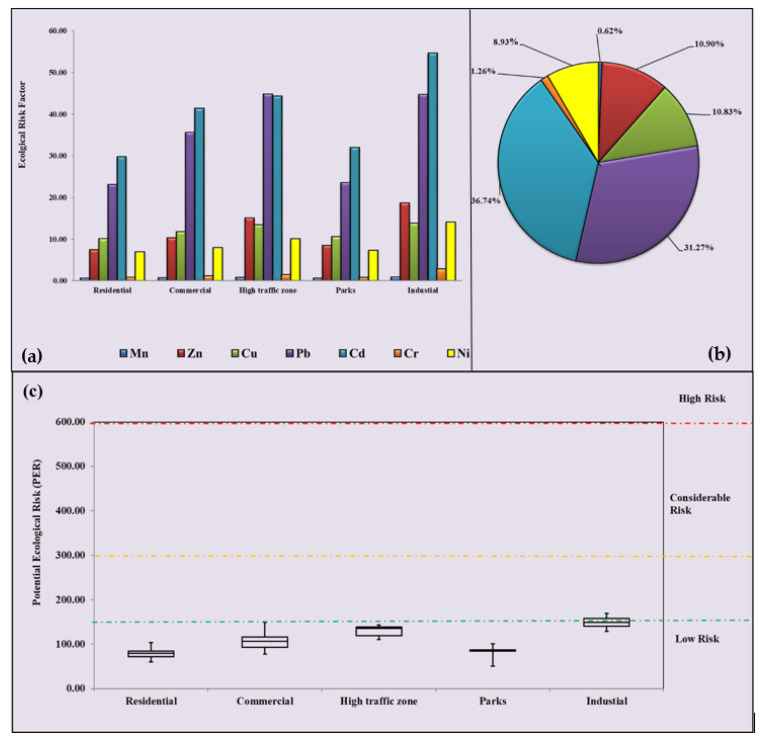
(**a**) Ecological risk factors (E_r_) of individual HMs in different functional areas; (**b**) relative percentage contributions of heavy metals to the potential ecological risk index (PER) in Lucknow; (**c**) box plot of potential ecological risk (PER). The box plot is outlined by the 25th and 75th percentile values, whiskers indicate the 10th and 90th percentile values, and dots show the 5th and 95th percentile outliers. The solid black line within the box corresponds to the 50th percentile value (or the median).

Figure 5a–c shows the ecological risk factors (E_r_) of Mn, Zn, Cu, Pb, Cd, Cr, and Ni and the potential ecological risk index of road dust particles from different functional areas of Lucknow. The average E_r_ value of Cd represents a moderate ecological risk (40 ≤ E_r_ ≤ 80) in industrial areas (54.58), high-traffic zones (44.28), and commercial areas (41.29), and a low ecological risk in residential and park areas (E_r_ < 40). Pb is in the moderate ecological risk category in high-traffic zones and industrial areas and the low ecological risk category in other functional areas. Mn, Zn, Cu, Cr, and Ni are in the low ecological risk category in all functional areas. The individual ecological risk of HMs in the study area increased in the order of Mn < Cr < Ni < Cu < Zn < Pb < Cd. Figure 5b shows that Cd contributed the highest ecological risk (36.74%) to PER followed by Pb (31.27%),Zn (10.93%), Cu (10.83%), Ni (8.93%), Cr (1.26%), and Mn (9.62%) in all five different functional areas. Therefore, it can be concluded that Cd, Pb, Zn, Cu, and Ni mainly cause the potential ecological risk of HMs in the road dust, and contribute almost 88.67% to the total PER in all different functional areas. Figure 5c shows that the PER values for all functional areas are in the low-risk category (PER < 150).

#### 3.5.2. Human Health Risk Assessment

- Non-carcinogenic risk assessment

Hazard quotient (HQ) and hazard index (HI) values were calculated as measures of non-carcinogenic (Fe, Mn, Zn, Cu, Pb, Cd, As, Cr, Ni) health risk for children and adults individually in all five functional areas and are shown in Table 8. It is seen from the results of non-carcinogenic risk that ingestion is the major exposure pathway for residents in all five functional areas for all studied HMs. Although the HQ and HI values of HMs were less than 1 (safe limit), HMs such as Pb Cr, Fe, and Mn had values near 1 for various exposure routes, in all functional areas, indicating that these HMs may cause non-carcinogenic risk for residents in the near future if anthropogenic activities continue to exceed their pollution levels. The value of combined HI for all HMs across all exposure pathways (i.e., cumulative HI) [31,36] was higher than the safe limit value of 1 for children in industrial (1.86), high-traffic (1.47), and commercial areas (1.17), suggesting significant potential risk. Overall, the HI values for all studied HMs were found in the decreasing order of Pb > Mn > Fe > Cr > Cu > As > Ni > Cd > Zn for both children and adults.

- Carcinogenic risk assessment

Table 9 shows the carcinogenic risk in the form of carcinogenic risk values for children and adults via three main pathways in all five studied functional areas of Lucknow for four HMs, i.e., Cd, As, Cr and Ni. The decreasing order of carcinogenic risk for the three studied pathways is as follows: CR_ingestion_ > CR_dermal_ > CR_inhalation_. The carcinogenic risk values for Cd, As, Cr, and Ni through the ingestion route were higher or equal to 1 × 10^−6^. The carcinogenic risk values for inhalation and dermal contact exposure routes were below the maximum permissible limit of 1.0 × 10^−6^, suggesting that the carcinogenic risk from these two pathways could be neglected. The LCR values were also higher than 1 × 10^−6^ for all studied HMs in both children and adults in all functional areas, indicating the possibility of cancer development in children and adults. In industrial areas, the carcinogenic risk values of Cr (1.61 × 10^−4^) and Ni (1.28 × 10^−4^) in children were higher than 1 × 10^−4^, indicating the development of cancer risk via these two metals in children. The results also reveal that the cancer risk appears to be higher for children compared to adults in all different functional areas. Cumulative LCR [31,36] values for children ranged from 0.000126 in residential areas to 0.000322 in industrial areas with an overall average value of 0.000187. For adults, cumulative LCR values were 0.0000541 in residential areas and 0.000138 in the industrial areas with an overall average value of 0.0000804. This suggests that children experience a higher cancer-causing risk compared to adults because of the HM-enriched dust exposure in Lucknow. The results reveal that the cancer risk exceeds the threshold value of 1 × 10^−4^–1 × 10^−6^, especially in children and industrial areas. Ni is the largest contributor to LCR in residential, commercial, high-traffic, and park areas, while Cr dominates in industrial areas.

## 4. Discussion

The present study shows that among the nine studied HMs, the mean concentrations of Zn, Pb, Cu, Mn, Ni, and Cd were higher and Fe and Cr were lower than the Indian natural soil background values. This shows that anthropogenic sources contribute a significant amount of these HMs to the road dust of the urban environment of Lucknow. Results from pollution indices(PI, IPI, and I_geo_) reveal that the road dust of Lucknow is highly contaminated with Zn, Pb, Cu, Ni, and Cd in almost all the five functional areas. Industrial and high-traffic areas show high contamination levels as compared to other functional areas. This may be mainly due to the industrial activities, vehicular activities, solid waste dumping, and construction and demolition activities in the city. Similar observations were also found in previous studies carried out by Roy et al. [5] in Delhi, Wei et al. [53] in China and Gope et al. [54] in Asansol, India. Zn, Pb, Cu, and Mn are mainly associated with tires, brake pads, brake linings, and vehicular exhausts [4,55]. Zn, in the form of zinc oxide, is used as an activator in the vulcanization process of tires, which converts soft, sticky rubber into a more stable component, whereas Cu is used in metallic and semi-metallic brake pads to improve the braking performance because of its comprehensive temperature ranges. Zn is also produced from galvanized metal parts, paint, and pigments, as well as brake and tire wear [5,7,56]. Lubricant oil, gasoline (tetraethyl) and paints (lead chromate, PbCrO_4_) are the main sources of Pb in road dust. Soil re-suspension also contributes Pb to road dust due to its previous deposition [3,57]. Construction and demolition activities also contribute Mn and Pb to road dust. Yellow and white paint marking in roads releases Pb, Cd, and Cr in road dust after abrasion. Cr plating is used to protect vehicular components from corrosion and wear and tear, whereas Ni is used in electric vehicle (EV) and engine batteries as well as automotive paints, which release these metals in road dust. Ni primarily arises from the combustion of fossil fuels and oil as well as solid waste burning near pavement areas [1,4]. The cement, leather, galvanizing, metal-based steel fabrication and processing, battery- and paint-related industries, and repairing and servicing workshops, also release these three metals into environments around the study area [8]. On the other hand, anthropogenically, Fe is produced mainly from vehicular non-exhaust sources such as brake and tire wear [55]. Fe and Cr also arise in road dust via lithogenic sources during soil resuspension. Cd and As are mainly produced from fossil fuel burning activities, but paint, agro-based, paper, pulp product electroplating, and pesticide-related industries also release these metals into the study areas.

The center, southwestern, and northeastern zones showed the highest HM distribution. This may be due to the city’s center and southwestern zones being highly enriched with almost all the heavy metals. This is due to the city’s central zone having ahigh population density and various major amenities such as educational centers, major commercial centers, administrative centers, coaching institutes, and government and private offices, which makes this zone busy with high vehicular traffic. The increasing trend of two-wheelers and three-wheelers (Autos/Tampos/Vikrams) produce more HMs in the central zone of the city. Encroachment of nearby roads by local shopkeepers, unplanned and illegal parking on nearby roads, and the poor traffic system worsen the situation. Traffic jams, frequent braking and start–stop patterns, congested roads, and nearby buildings make the situation even worse and HMs produced from vehicles easily accumulate in these areas because of the low dispersal rate. Industries making agricultural tools, chemicals, pharmaceuticals, precision instruments, and bicycle parts, and engineering, electrical, and hardware factories in the industrial areas such as Talkatora emit a large number of HMs such as Pb, Cd, Cr, Ni and As in this zone. Apart from these, the city’s small-scale and cottage industries such as handicrafts and textiles are concentrated in Chowk, Aminabad, Bashiratganj, etc., also found in the central zone of the city. The southwestern zone has facilities such as an airport, railway station, interstate bus stand, and some famous commercial centers. Hundreds of small and large hotels, restaurants, motels, and Dharamshala found in this area also make this region crowded with locals and visitors and different types of vehicles during the day and night. Apart from this, two main industrial areas also fall under this zone, i.e., Amausi and Sarojini Nagar, which are the home of many medium- and small-scale industries such as battery, plastic, ceramic, and paint production, which release substantial amounts of metals such as Mn, Fe, Cr, Ni, Cu, Cd into the urban environment. Southeastern and north locations mainly have residential establishments, a large cantonment area (prohibited for public use), Nawabganj wildlife sanctuary, and parks. The population and vehicular density are lower compared to the other areas, so this zone has a lower concentration range for HMs, especially Cd, Cu, Zn, Pb, and As, which are produced from industrial and vehicular activities.

The ecological risk assessment results show that Cd, Pb, Zn, Cu and Ni contributed the most to ecological risk in all studied functional areas. Similar results were shown in other studies by Gupta et al. [4], Roy et al. [5], and Taiwo et al. [31]. The results reveal that 96.88% of locations out of 64 sampling locations are in the low-risk category, and 3.13% of locations are in the moderate-risk category. Although the PER of HMs studied in the road dust of five different functional areas shows that the risk is at a low level in all studied functional areas, some industrial locations also show a moderate risk. This can be attributed to high-traffic and industrial activities in these locations. Human health risk assessments revealed that ingestion is the primary pathway of HM exposure in both children and adults. Most of the HMs enter into the human body via ingestion as we consume xenobiotic compounds (solids and liquids) and HM-contaminated food, water, and liquids on a daily basis. Previous investigations by Gupta et al. [4], Parveen and Aris [29], and Taiwo et al. [31] revealed similar results for Dehradun city, Uttarakhand, Rawang city, Malaysia, and Osun state, southwestern Nigeria, respectively. Industrial, commercial, and high-traffic areas showed a high non-carcinogenic health risk for children. It is also alarming to note that residential and park areas also had values near 1 for children due to HM contamination in Lucknow, indicating the possibility of non-carcinogenic health risks in these two areas as well. Carcinogenic risk results revealed that due to Cd, As, Cr, and Ni, there is a possibility of cancer risk via the ingestion route in both age groups. Children are more susceptible to both non-carcinogenic and cancer risk. This could be due to their outside activities, for example frequent hand licking, eating behavior, and eating disorders such as pica disorder [31,34]. In cities like Lucknow, many poor young children work outdoors as street vendors selling different items, especially in the high-traffic areas and commercial zones, and other workers on the roadside are exposed more towards this risk. School-going children are also at high risk as they are exposed to this pollution on a daily basis [21].

## 5. Conclusions

The present study investigated the concentration levels of HMs in road dust, their sources and spatial distribution, and how these metals cause non-carcinogenic and carcinogenic risks in two age groups. The concentration results reveal that almost all HMs exceed the background INSB limits, which means that HMs accumulated in the road dust via various anthropogenic and natural sources. In all functional areas, vehicular sources turn out to be the most significant anthropogenic source of HMs Mn, Zn, Cu, and Pb, while industrial activities mainly contribute to the HMs Ni, Cr, Cd, and As in the road dust. Almost all studied HMs were identified in all studied functional areas because of intermixing and overlapping of different functional areas. The central zone, its adjacent areas, and the southwestern zone of the city, which possess various commercial, residential and industrial areas, experience worse road dust quality. Most of these zones are already overflowing with people and vehicles. Now the city is expanding to accommodate a growing population by creating new residential and commercial zones on the outer periphery of the city and north and southeastern zones where the HMs show lower concentration range in spatial distribution. High vehicular density, roadside encroachment by street vendors and small shopkeepers, and roadside illegal parking stands are common in Lucknow, which create traffic jams in the city and worsen environmental conditions. Continuous construction and demolition as well as the close proximity of buildings toward roads decrease the dispersal rate of dust, allowing HMs to accumulate more in road dust. Large populations using public transport e.g., city buses, and three-wheeler (Auto/Tampos) rickshaws to travel from one place to another in the city are more exposed because dust can directly enter through the nose and mouth as doors and windows are usually open in these vehicles. The increasing trend of using two-wheelers shows that more people are exposed to polluted road dust. The habit of not wearing helmets and masks will worsen the situation more for the rider of two-wheelers. Pedestrians, traffic policemen, street vendors, outdoor workers, and auto drivers are at high risk as they spend long hours outdoors and in areas with high levels of HM-enriched dust. The current findings show that HMs can produce both non-carcinogenic and carcinogenic risks for the people of Lucknow, especially children who are more susceptible to these risks. It is necessary that we must constantly monitor the concentration, bioavailability, distribution, and health effects of HMs in various environmental components in Lucknow city. There is a need to strictly monitor the level of HMs in liquid and solid food items as ingestion was shown to be the major exposure pathway. Masks should be prescribed for people working in high-traffic and industrial areas on a daily basis. As demolition and construction activities play a huge role in creating dust pollution, the government need to take strict preventive measures to reduce this. Management plans and strategies should concentrate on highly polluted and crowded areas of the city.

## Figures and Tables

**Table 1:** Sampling locations representing five different functional areas (i.e., residential, commercial, high-traffic areas, parks, and industrial areas).

S.No.	Location	Functional Areas	Latitude	Longitude	S.No.	Location	Functional Areas	Latitude	Longitude
**1**	Aishbagh	Residential	26.84	80.92	**33**	SmritiUpvan	Park	26.79	80.92
**2**	Krishna Nagar	Residential	26.79	80.88	**34**	Jonal Park	Park	26.79	80.91
**3**	Banthara	Residential	26.69	80.84	**35**	Malihabaad	Park	26.92	80.71
**4**	Aliganj	Residential	26.90	80.95	**36**	Transport Nagar	Commercial	26.78	80.89
**5**	Niralanagar	Residential	26.88	80.94	**37**	Mawaiya	Commercial	26.83	80.91
**6**	Khadra	Residential	26.88	80.91	**38**	Aminabad	Commercial	26.84	80.92
**7**	Purania	Residential	26.90	80.94	**39**	Residency	Commercial	26.86	80.95
**8**	Kapoorthala	Residential	26.88	80.94	**40**	Nishatganj	Commercial	26.83	80.90
**9**	Jankipuram	Residential	26.92	80.95	**41**	Sikandarbagh/Nbri	Commercial	26.86	80.95
**10**	Up RSAC	Residential	26.91	80.96	**42**	Hazaratganj	Commercial	26.86	80.95
**11**	Balaganj	Residential	26.88	80.88	**43**	Vidhansabha	Commercial	26.84	80.94
**12**	Thakurganj	Residential	26.88	80.89	**44**	Daliganj	Commercial	26.87	80.93
**13**	Wazirganj	Residential	26.85	80.92	**45**	Hussainganj	Commercial	26.84	80.93
**14**	Gomatinagar	Residential	26.86	81.02	**46**	Chowk	Commercial	26.86	80.90
**15**	Vipulkhand	Residential	26.84	80.99	**47**	Bara Imambara	Commercial	26.87	80.91
**16**	Indiranagar	Residential	26.88	80.99	**48**	Yahiyaganj	Commercial	26.86	80.91
**17**	Mahanagar	Residential	26.88	81.00	**49**	Patrakarpuram	Commercial	26.85	81.00
**18**	Omax City	Residential	26.76	80.92	**50**	Lekhraj Market	Commercial	26.87	80.97
**19**	Golf City	Residential	26.79	81.04	**51**	Hal	Commercial	26.87	80.99
**20**	Rajajipuram	Residential	26.85	80.88	**52**	Bbau	Commercial	26.77	80.92
**21**	Ashiyana	Residential	26.80	80.92	**53**	Gole Market	Commercial	26.87	80.96
**22**	Vikas Nagar	Residential	26.90	80.96	**54**	Dubbagga	Commercial	26.87	80.86
**23**	Uthretiya	Residential	26.79	80.92	**55**	Amausi	Industrial	26.76	80.87
**24**	Kukrail	Park	26.91	80.98	**56**	Chinhat	Industrial	26.88	81.04
**25**	Janeshwar Mishra Park	Park	26.84	81.00	**57**	Sarojini Nagar	Industrial	26.75	80.86
**26**	Nimbu Park	Park	26.87	80.91	**58**	Talkatora	Industrial	26.83	80.89
**27**	Hathi Park	Park	26.87	80.92	**59**	Chabagh Station	High-Traffic Zone	26.83	80.92
**28**	Ramabai Ambedkar Park	Park	26.76	80.92	**60**	Alambagh Chauraha	High-Traffic Zone	26.83	80.91
**29**	Lohia Park	Park	26.85	80.98	**61**	Kaiserbagh	High-Traffic Zone	26.86	80.92
**30**	Ambedkar Park	Park	26.85	80.98	**62**	Polytechnique Chauraha	High-Traffic Zone	26.87	81.00
**31**	Zoo	Park	26.85	80.95	**63**	Barlington Chauraha	High-Traffic Zone	26.84	80.94
**32**	Kanshiram Eco Garden	Park	26.81	80.92	**64**	Engineering College Chauraha	High-Traffic Zone	26.91	80.94

**Table 2:** Pollution index (PI), integrated pollution index (IPI), ecological risk factor (ER), and potential ecological risk (PER) classification.

Pollution Index (PI)	Classification	Integrated Pollution Index (IPI)	Classification	Ecological Risk Factor (E_r_)	Classification	PER	Classification
PI ≤ 1	Low pollution level	IPI ≤ 1	Low level of pollution	E_r_ < 40	Low risk	PER < 150	Low risk
1 ≤ PI ≤ 3	Moderate pollution level	1 < IPI ≤ 2	Moderate level of pollution	40 ≤ E_r_ < 80	Moderate risk	150 ≤ PER < 300	Moderate risk
		2 < IPI > 5	High level of pollution	80 ≤ E_r_ < 160	Considerable risk	300 ≤ PER < 600	Considerable risk
PI ≥ 3	High pollution level	IPI > 5	Extremely high level of pollution	160 ≤ E_r_ < 320	High risk	PER > 600	High risk
				E_r_ > 320	Very high risk		

PI and IPI values were adopted from Wan et al. [38]. ER and PER classification is based on Hakanson [44].

**Table 3:** Exposure factor values used in average daily intake (ADI) estimation for non-carcinogenic risk for children and adults.

Parameter	Definition	Unit	Value	
			Children	Adult
C	Concentration of metal	mg·kg^−1^	-	-
IngR	Ingestion rate	mg·day^−1^	100	200
EF	Exposure frequency	days·year^−1^	350	350
ED	Exposure duration	years	6	24
BW	Body weight	kg	16.2	61.8
AT	Average time (non-carcinogenic)	days	ED × 365	ED × 365
InhR	Inhalation rate	m^3^·day^−1^	72 × 365	72 × 365
PEF	Particle emission factor	m^3^·kg^−1^	7.6	20
SA	Exposed skin area	cm^2^	1.36 × 10^9^	1.36 × 10^9^
SL	Skin adherence factor	mg·cm^−2^·day^−1^	2800	5700
ABS	Dermal absorption factor	-	0.20	0.07

Adapted from the US Environmental Protection Agency (USEPA) [46].

**Table 4:** Toxic response factor, reference doses (R_f_D), and cancer slope factor (CSF) for heavy metals.

Heavy Metals	Toxic Response Factors	Reference Dose (R_f_D, mg·kg^−1^·day^−1^)	Cancer Slope Factor (CSF, mg·kg^−1^·day^−1^)
Fe	-	0.7	-
Mn	1	0.014	-
Zn	1	0.3	-
Cu	5	0.04	-
Pb	5	0.035	-
Cd	30	0.001	15
As	10	0.003	1.5
Cr	2	0.003	0.5
Ni	5	0.02	0.91

R_f_D and CSF values were adopted from the US Environmental Protection Agency (USEPA) [49].

**Table 5:** Heavy metal concentrations (μg·g^−1^) in road dust samples from urban Lucknow across different functional areas.

Functional Areas		Fe	Mn	Zn	Cu	Pb	Cd	As	Cr	Ni
Residential (N = 23)	Average ± SD	9620.07 ± 3317	297.12 ± 66.23	162.92 ± 49.53	113.57 ± 14.46	60.54 ± 16.17	0.89 ± 0.06	6.94 ± 1.12	45.22 ± 110.93	37.92 ± 13.63
	Range	5100.00–16235.50	202.36–425.25	85.52–312.25	90.15–145.50	34.40–90.56	0.81–1.00	5.01–8.78	25.52–65.98	15.15–60.85
Commercial (N = 19)	Average ± SD	13,042.19 ± 47,673	44.05 ± 109.48	226.74 ± 114.16	132.78 ± 22.33	93.28 ± 21.19	1.24 ± 0.13	7.98 ± 1.26	63.44 ± 14.78	43.89 ± 11.88
	Range	5153.45–25,458.85	202.02–550.45	115.23–485.45	97.20–170.15	55.85–140.45	1.05–1.56	5.59–9.89	36.85–92.48	30.24–70.25
High traffic (N = 06)	Average ± SD	17,432.90 ± 4152.02	406.97 ± 87.56	332.91 ± 74.06	151.13 ± 12.19	117.32 ± 13.70	1.33 ± 0.10	8.91 ± 1.32	82.53 ± 9.73	55.35 ± 7.61
	Range	10,657.32–22,625.25	330.20–550.50	215.55–400.45	139.85–165.45	100.22–130.45	1.20–1.45	7.21–11.25	68.62–95.12	45.45–65.11
Parks (N = 12)	Average ± SD	10,671.28 ± 3027.68	300.94 ± 32.11	186.83 ± 19.97	119.37 ± 8.86	61.60 ± 10.62	0.96 ± 0.31	7.02 ± 1.32	42.49 ± 10.84	40.09 ± 5.79
	Range	5556.42–15,200.32	245.52–350.45	155.33–220.45	112.45–145.20	51.36–90.15	0.12–1.15	5.31–9.21	25.52–65.48	32.50–51.50
Industrial (N = 04)	Average ± SD	24,326.21 ± 1745.78	444.62 ± 118.78	412.93 ± 161.17	155.39 ± 18.01	117.15 ± 11.13	1.64 ± 0.08	11.36 ± 0.89	160.72 ± 13.45	77.58 ± 8.44
	Range	22,425.82–26,258.40	315.55–565.32	215.50–594.23	135.85–175.12	105.66–130.22	1.56–1.75	10.29–12.39	140.78–170.17	65.55–85.15

**Table 6:** Heavy metal concentrations (mg·kg^−1^) in road dust samples from different regions of India and the world.

City (Country) and Recommended Body	Fe	Mn	Zn	Cu	Pb	Cd	As	Cr	Ni	Reference
**Lucknow, India**	**16,368.15**	**374.15**	**289.85**	**139.66**	**97.34**	**1.29**	**8.82**	**87.29**	**54.23**	**Present study**
Delhi, India	27047	699.2	263.7	168.7	128.7	-	-	170.8	37.2	Rajaram et al. [1]
Dhanbad, India	67,700	1500	224	53.6	128	-	17.5	45.2	22	Masto et al. [35]
Kolkata, India	40,334	743	289	92	128	-	-	164	36	Kolakkandi et al. [36]
Dehradun, India	12,509.51	315.58	217.10	80.41	138.77	-	4.40	46.75	35.38	Bisht et al. [3]
Xi’an, China	-	337.6	169.2	46.6	97.4	-	-	177.5	29.3	Lu et al. [50]
Dhaka (Bangladesh)	-	261.53	239.1	49.8	18.9	11.6	8.1	144.3	37.1	Rehman et al. [18]
Dezful (Iran)	-	-	224	51	54	0.4	3	44	46	Sadeghdoust et al. [32]
Lahore (Pakistan)	-	-	1053	116	230	5.17	7.51	194	53.6	Rehman et al. [13]
Kabul (Afghanistan)	-	252.95	122.51	43.63	28.69	1.16	-	38.40	66.41	Jadoon et al. [30]
Concentration in Indian natural soil	30,890	527	22.1	56.5	13.1	0.90	-	114	27.7	Kuhad et al. [39] and Gowd et al. [40]
Continental upper crust	32,015	209	52	14.3	17	0.102	2.0	35	18.6	Wedepohl [52]

**Table 7:** PCA loadings of variables of significant principal components.

Parameters	PC1	PC2	PC3
Fe	0.384	**0.715**	0.319
Mn	**0.709**	0.547	0.019
Zn	**0.661**	0.551	0.322
Cu	**0.841**	0.360	0.251
Pb	**0.838**	0.257	0.411
Cd	0.513	0.294	**0.643**
As	0.132	0.213	**0.901**
Cr	0.387	**0.621**	0.592
Ni	0.321	**0.808**	0.271
Eigen Value	3.029	2.480	2.089
% of Variance	33.661	27.550	23.208
Cumulative %	33.661	61.211	84.419

Extraction method: principal component analysis; rotation method: varimax with Kaiser normalization.

**Table 8:** Summary of hazard quotient (HQ) and hazard index (HI) values of HMs in road dust for five different functional areas.

Heavy Metals	Type of Exposure	Different Functional Areas									
		Residential		Commercial		High-Traffic Areas		Parks		Industrial	
		Children	Adults	Children	Adults	Children	Adults	Children	Adults	Children	Adults
Fe	HQ_Ingestion_	0.176	0.0188	0.238	0.0255	0.318	0.0341	0.195	0.0209	0.444	0.0476
	HQ_Inhalation_	0.00000491	0.00000277	0.00000666	0.00000375	0.00000890	0.00000502	0.00000545	0.00000307	0.0000124	0.00000700
	HQ_Dermal_	0.000492	0.00000751	0.000667	0.000102	0.000892	0.000136	0.000546	0.0000833	0.00124	0.000190
	HI	**0.176**	**0.0189**	**0.239**	**0.0256**	**0.319**	**0.0343**	**0.195**	**0.0210**	**0.446**	**0.0478**
Mn	HQ_Ingestion_	0.271	0.0291	0.314	0.0337	0.372	0.0398	0.275	0.0294	0.406	0.0435
	HQ_Inhalation_	0.00000758	0.00000428	0.00000878	0.00000495	0.0000104	0.00000586	0.00000768	0.00000433	0.0000113	0.00000640
	HQ_Dermal_	0.000760	0.000116	0.000880	0.000134	0.00104	0.000159	0.000770	0.000117	0.00114	0.000174
	HI	**0.272**	**0.0292**	**0.315**	**0.0338**	**0.373**	**0.0400**	**0.276**	**0.0296**	**0.407**	**0.0437**
Zn	HQ_Ingestion_	0.00694	0.000744	0.00966	0.00104	0.0142	0.00152	7960.00	0.000853	0.0176	0.00189
	HQ_Inhalation_	0.000000194	0.000000109	0.000000270	0.000000152	0.000000396	0.000000224	0.000000222	0.000000125	0.000000492	0.000000277
	HQ_Dermal_	0.0000194	0.00000297	0.0000271	0.00000413	0.0000397	0.00000607	0.0000223	0.00000340	0.0000493	0.00000752
	HI	**0.00696**	**0.000747**	**0.00969**	**0.00104**	**0.0142**	**0.00152**	**0.00798**	**0.000857**	**0.0176**	**0.00189**
Cu	HQ_Ingestion_	0.0363	0.00389	0.0424	0.00455	0.0483	0.00518	0.0382	0.00409	0.0497	0.00532
	HQ_Inhalation_	0.00000101	0.000000572	0.00000119	0.000000669	0.00000135	0.000000761	0.00000107	0.000000601	0.00000139	0.000000783
	HQ_Dermal_	0.000102	0.0000155	0.000119	0.0000181	0.000135	0.0000207	0.000107	0.0000163	0.000139	0.0000212
	HI	**0.0364**	**0.00391**	**0.0426**	**0.00457**	**0.0484**	**0.00520**	**0.0383**	**0.00410**	**0.0498**	**0.00534**
Pb	HQ_Ingestion_	0.221	0.0237	0.341	0.0365	0.429	0.0459	0.225	0.0241	0.428	0.0459
	HQ_Inhalation_	0.00000618	0.00000348	0.00000952	0.00000537	0.0000120	0.00000675	0.00000629	0.00000355	0.0000120	0.00000674
	HQ_Dermal_	0.000619	0.0000945	0.000954	0.000146	0.00120	0.000183	0.000630	0.0000962	0.00120	0.000183
	HI	**0.222**	**0.0238**	**0.342**	**0.0367**	**0.430**	**0.0461**	**0.226**	**0.0242**	**0.429**	**0.0460**
Cd	HQ_Ingestion_	0.0114	0.00122	0.0158	0.00170	0.0170	0.00182	0.0122	0.00131	0.0209	0.00224
	HQ_Inhalation_	0.000000318	0.000000179	0.000000442	0.000000250	0.000000475	0.000000268	0.000000342	0.000000193	0.000000585	0.000000330
	HQ_Dermal_	0.0000319	0.00000487	0.0000443	0.00000677	0.0000476	0.00000726	0.0000343	0.00000524	0.0000586	0.00000895
	HI	**0.0114**	**0.00123**	**0.0159**	**0.00170**	**0.0170**	**0.00183**	**0.0123**	**0.00132**	**0.0210**	**0.00225**
As	HQ_Ingestion_	0.0385	0.00413	0.0340	0.00364	0.0380	0.00407	0.0299	0.00321	0.0484	0.00518
	HQ_Inhalation_	0.00000108	0.000000607	0.000000950	0.000000536	0.00000106	0.000000598	0.000000836	0.000000472	0.00000135	0.000000762
	HQ_Dermal_	0.000108	0.0000165	0.0000952	0.0000145	0.000106	0.0000162	0.0000838	0.0000128	0.000135	0.0000207
	HI	**0.0386**	**0.00414**	**0.0341**	**0.00366**	**0.0381**	**0.00408**	**0.0300**	**0.00322**	**0.0485**	**0.00521**
Cr	HQ_Ingestion_	0.120	0.0128	0.162	0.0174	0.211	0.0226	0.109	0.0116	0.411	0.0440
	HQ_Inhalation_	0.00000335	0.00000189	0.00000453	0.00000256	0.00000590	0.00000333	0.00000304	0.00000171	0.0000115	0.00000648
	HQ_Dermal_	0.000335	0.0000512	0.000454	0.0000693	0.000591	0.0000902	0.000304	0.0000464	0.00115	0.000176
	HI	**0.120**	**0.0129**	**0.163**	**0.0175**	**0.212**	**0.0227**	**0.109**	**0.0117**	**0.412**	**0.0442**
Ni	HQ_Ingestion_	0.0242	0.00260	0.0281	0.00301	0.0354	0.00379	0.0256	0.00275	0.04962	0.00531
	HQ_Inhalation_	0.000000677	0.000000382	0.000000784	0.000000442	0.000000989	0.000000557	0.000000716	0.000000404	0.00000139	0.000000781
	HQ_Dermal_	0.0000679	0.0000104	0.0000786	0.00000120	0.0000991	0.0000151	0.0000718	0.0000110	0.000139	0.0000212
	HI	**0.0243**	**0.00261**	**0.0281**	**0.00302**	**0.0355**	**0.00381**	**0.0257**	**0.00276**	**0.0497**	**0.00534**
Cumulative HI		**0.896**	**0.0962**	**1.17**	**0.126**	**1.47**	**0.158**	**0.908**	**0.0974**	**1.86**	**0.199**

**Table 9:** Carcinogenic risk of HMs in road dust for five different functional areas.

Heavy Metals	Type of Exposure	Different Functional Areas									
		Residential		Commercial		High-Traffic Areas		Parks		Industrial	
		Children	Adults	Children	Adults	Children	Adults	Children	Adults	Children	Adults
Cd	CR_Ingestion_	0.0000146	0.00000628	0.0000204	0.00000873	0.0000218	0.00000936	0.0000157	0.00000675	0.0000269	0.0000115
	CR_Inhalation_	0.000000000409	0.000000000923	0.000000000569	0.00000000128	0.000000000610	0.00000000138	0.000000000440	0.000000000990	0.000000000752	0.00000000170
	CR_Dermal_	0.000000410	0.0000000250	0.0000000570	0.0000000348	0.0000000611	0.0000000373	0.0000000441	0.0000000269	0.0000000754	0.0000000460
	LCR	**0.0000147**	**0.00000630**	**0.0000204**	**0.00000876**	**0.0000219**	**0.00000940**	**0.0000158**	**0.00000678**	**0.0000270**	**0.0000116**
As	CR_Ingestion_	0.00000380	0.00000163	0.00000437	0.00000187	0.00000488	0.00000209	0.00000385	0.00000165	0.00000622	0.00000267
	CR_Inhalation_	0.000000000106	0.000000000240	0.000000000122	0.000000000276	0.000000000136	0.000000000308	0.000000000108	0.000000000243	0.000000000174	0.000000000382
	CR_Dermal_	0.0000000106	0.00000000650	0.0000000122	0.00000000748	0.0000000137	0.00000000835	0.0000000108	0.00000000658	0.0000000174	0.0000000106
	LCR	**0.00000381**	**0.00000164**	**0.00000438**	**0.00000188**	**0.00000489**	**0.00000210**	**0.00000386**	**0.00000166**	**0.00000624**	**0.00000268**
Cr	CR_Ingestion_	0.0000451	0.0000193	0.0000633	0.0000271	0.0000823	0.0000353	0.0000424	0.0000182	0.000160	0.0000687
	CR_Inhalation_	0.00000000126	0.00000000284	0.00000000177	0.00000000399	0.00000000230	0.00000000519	0.00000000118	0.00000000267	0.00000000448	0.0000000101
	CR_Dermal_	0.000000126	0.0000000771	0.000000177	0.000000108	0.000000230	0.000000141	0.000000119	0.0000000725	0.000000449	0.000000274
	LCR	**0.0000452**	**0.0000194**	**0.0000634**	**0.0000272**	**0.0000825**	**0.0000354**	**0.0000425**	**0.0000182**	**0.000161**	**0.0000690**
Ni	CR_Ingestion_	0.0000623	0.0000267	0.0000721	0.0000309	0.0000910	0.0000390	0.0000659	0.0000282	0.000128	0.0000547
	CR_Inhalation_	0.00000000174	0.00000000393	0.00000000202	0.00000000455	0.00000000254	0.00000000573	0.00000000184	0.00000000415	0.00000000356	0.00000000804
	CR_Dermal_	0.000000175	0.000000107	0.000000202	0.000000123	0.000000255	0.000000156	0.000000185	0.000000113	0.000000357	0.000000218
	LCR	**0.0000625**	**0.0000268**	**0.0000723**	**0.0000310**	**0.0000912**	**0.0000392**	**0.0000661**	**0.0000284**	**0.000128**	**0.0000549**
Cumulative LCR		**0.000126**	**0.0000541**	**0.000160**	**0.0000688**	**0.000200**	**0.0000861**	**0.000128**	**0.0000550**	**0.000322**	**0.000138**

## Data Availability

The datasets used and/or analyzed during the current study are available from the corresponding author upon reasonable request.

## List of Abbreviation

ADI	Average Daily Intake
CR	Cancer Risk
CSF	Cancer-Slope Factor
E_r_	Ecological Risk Factor
HI	Hazard Index
HM	Heavy Metal
HMs	Heavy Metals
HQ	Hazard Quotient
HRA	Health Risk Assessment
ICPMS	Inductively Coupled Plasma-Mass Spectroscopy
IDW	Inverse Distance Weighting
INSB	Indian Natural Soil Background
IPI	Integrated Pollution Index
LCR	Lifetime Cancer Risk
PCA	Principal Component Analysis
PER	Potential Ecological Risk
PI	Pollution Index
Tr	Toxic-Response Factor
UCC	Upper Continental Crust
